# Attitudes and beliefs regarding cannabis use during pregnancy compared to alcohol and tobacco: a nationwide survey of U.S. women of childbearing age

**DOI:** 10.1186/s42238-026-00466-0

**Published:** 2026-07-04

**Authors:** SG. Casavant, N. Kilembe, S. Kinsey, N. Shook

**Affiliations:** 1 Elisabeth DeLuca School of Nursing, University of Connecticut, 231 Glenbrook Rd U-4026, Storrs, CT 06269 USA; 2 Department of Pediatrics, University of Connecticut School of Medicine, Farmington, CT USA

**Keywords:** Cannabis, Pregnancy, Alcohol, Tobacco, Perceptions, Survey

## Abstract

**Background:**

Cannabis use is increasing, even during pregnancy. The purpose of this study was to investigate how women of childbearing age perceive the safety of cannabis use during pregnancy compared to alcohol and tobacco use.

**Methods:**

Cloud Research recruited a national U.S. cohort of women of childbearing age (*N* = 622) to complete an anonymous online survey assessing cannabis, alcohol, and tobacco use during pregnancy.

Validated tools measured perceptions of safety and risks to fetal, birth, and infant development. Data analyses included descriptive statistics, McNemar’s tests, and repeated-measures ANOVA with post-hoc comparisons.

**Results:**

The average age was 29.47 years (*SD* = 6.83, range: 18–42). Participants self-identified as White (65.9%, *n* = 410), Black or African American 28.1% (*n* = 175), Asian 5.9% (*n* = 37), American Indian or Alaska Native 3.9% (*n* = 24), or Other 4.7% (*n* = 29); 17.7% (*n* = 110) identified as Hispanic or Latino. Most participants identified as heterosexual (82.0%, *n* = 510). Among participants who had been pregnant (*N* = 351), 25.9% reported cannabis use during pregnancy, compared with 23.6% for tobacco and 8.2% for alcohol. The median frequency of prenatal cannabis use was twice weekly, with joints and blunts being the most common methods. Reported reasons for use included relief of nausea, anxiety, sleep disturbance, and pain. We used a five-point Likert scale to determine whether they thought cannabis, alcohol, and tobacco were safe to use during pregnancy, where 1 = *not at all safe*, and 5 = *completely safe*. Prenatal cannabis use was rated as safer (*M* = 3.85, *SD* = 1.46) than alcohol. (*M* = 4.86, *SD* = 0.54, *p* < .001) or tobacco (*M* = 4.79, *SD* = 0.63, *p* < .001).

**Conclusion:**

Findings indicate that women of childbearing age perceive cannabis as relatively safe during pregnancy, particularly for symptom management. These perceptions underscore the need for targeted education, accurate messaging, and consideration of the social, cultural, and emotional factors that influence substance use during pregnancy to safeguard maternal and fetal health.

## Background

Societal perceptions and use of cannabis products have changed significantly in the United States, as evidenced by the legalization of recreational cannabis in 24 U.S. states and Washington, D.C., as of 2024. With legalization, the use of cannabis products has increased markedly. For example, 3.7 million people over the age of 12 tried cannabis products for the first time in 2022, a 42% increase from the previous year. (Administration SAaMHS 2022) Of particular concern, this trend is also apparent in pregnant women. (Gnofam et al. 2020).

Both cigarette smoking and alcohol use during pregnancy are linked to higher chances of preterm birth, low birth weight, and poorer infant health outcomes. (Hamulka et al. 2018) Cannabis products are increasingly advertised as safe, natural, and effective remedies for common daily ailments, including pregnancy-related issues such as insomnia, pain, and morning sickness. (Dickson et al. 2018) Volkow et al. analyzed data from the U.S. National Survey on Drug Use and Health and reported significant increases in the prevalence and frequency of cannabis use among pregnant women from 2002 to 2017. (Volkow et al. 2019) In contrast, other authors analyzed data from the U.S. National Survey on Drug Use and Health from 2002 to 2019 and found no significant increase in the prevalence of cannabis dependence among pregnant women. (Alshaarawy and Vanderziel 2002) However, Ko and colleagues analyzed data from the U.S. National Survey on Drug Use from 2007 to 2012 and found that 18.1% of pregnant women met criteria for abuse or dependence based on DSM-IV criteria for cannabis abuse and dependence. (Ko et al. 2015; Association AP 2000).

Compared with well-established teratogens such as alcohol or tobacco, less is known about the potential adverse developmental effects of cannabis exposure during pregnancy. Researchers have found adverse or no adverse outcomes associated with cannabis exposure during pregnancy. For example, a recently published systematic review examined the association of cannabis product use during pregnancy with birth outcomes (i.e., preterm birth,, gestational age, health at delivery) in both rodent and human models. (Reck et al. 2025) Among studies focusing on preterm birth, ten reported a significant association between prenatal cannabis product use and an increased likelihood of preterm delivery, whereas 11 reported no association between prenatal cannabis exposure (PCE) and preterm delivery. Of the four studies that examined the overall length of gestation, two reported that a lower gestational age was associated with PCE, whereas two reported no association between PCE and gestational length. Overall, the review found that prenatal cannabis exposure was associated with lower birth weight in both humans and rodents, and there was some evidence that prenatal cannabis exposure was associated with lower gestational age and poorer infant health at birth. (Reck et al. 2025) Although most studies in that review had small sample sizes, another team updated their review and meta-analysis with a larger sample size (> 1.7 million) than their previous review, and they found that PCE was also associated with preterm birth, infants being small for gestational age and low birthweight with confidence in their findings improving from low in the original review to moderate in their more recent meta-analysis. (Lo et al. 2025) However, the conclusions were uncertain given methodological limitations, such as reliance on retrospective self-report of cannabis use without information on product type, frequency, timing, or dosage; limited use of objective exposure measures; and few longitudinal designs capable of capturing changes in use across pregnancy.

Knowledge of reasons for prenatal cannabis use is also limited. Multiple groups have reported that women use cannabis during pregnancy to cope with nausea, vomiting and pain (Brik et al. 2024; Chang et al. 2019), as well as sleep, depression, stress and anxiety. (Young-Wolff et al. 2024).

Furthermore, little is known about the perceived safety or risk of prenatal cannabis use. Foti et al. conducted 18 focus groups with 53 pregnant participants who used cannabis at prenatal care entry in California, where medical and recreational use is legal. (Foti et al. 2023) Several participants perceived prenatal cannabis use as safer than prescription or over-the-counter medications. Another group of authors extracted 151 original posts and 1,260 corresponding responses from an online cannabis forum called Ganja Mamas forum (https://community.whattoexpect.com/forums/ganja-mamas.html) to examine information-seeking behavior regarding cannabis use during pregnancy. (Lebron et al. 2022) They found that pregnant women on the forum primarily sought information related to legal issues in their geographical location, for example, drug-testing practices and involvement of child protective services. (Lebron et al. 2022) The gap between perceived safety and documented risk poses a significant challenge for public health. The authors of one integrative review found that health care providers did not communicate potential risks of using cannabis while pregnant, which led pregnant women to perceive it as relatively safe. (Bayrampour et al. 2019) McKenzie et al. conducted focus groups with 21 pregnant women about health behaviors during pregnancy in Columbus, Ohio, and found that distrust of health care providers was a salient theme. (McKenzie et al. 2022) The authors of a cross-sectional U.S. survey of 284 pregnant women indicated that two-thirds of pregnant women who use cannabis view it as helpful for symptom relief. (Satti et al. 2022) A nationwide study indicated that women who reported regular marijuana use, whether pregnant or not, were more likely to perceive it as having no risk compared to women who did not report marijuana use. (Malone et al. 2026).

Without clear evidence, communication, and supportive interventions, misconceptions will persist, and use may continue to rise. Against this background, the purpose of this study was to examine cannabis use among women of childbearing age and to compare the risks they perceived associated with cannabis, alcohol, and tobacco during pregnancy. We expected prenatal cannabis use to be perceived as safer than prenatal alcohol or tobacco use.

## Methods

We conducted an online cross-sectional survey of U.S. women of childbearing age. We received approval for our study protocol from the University of Connecticut Institutional Review Board (Protocol L24-0230). 

Participants completed an online survey administered via Qualtrics. A survey link was distributed to women of childbearing age in the United States through CloudResearch (Clarkston, MI), an online platform that provides access to diverse samples of U.S. adults. To ensure data quality, CloudResearch uses proprietary software to identify and exclude fraudulent, automated, or inattentive responses, including, but not limited to, multiple attempts made from the same Internet Protocol address.

Eligible participants were women 18 to 42 years of age, lived in the United States, and were proficient in the English language. An a priori power analysis using G*Power 3, (Faul et al. 2007) indicated that a minimum sample size of 492 was necessary to detect small to medium effects (f = 0.15, α = 0.05, power = 0.80) for between-group comparisons. To account for potential missing or unusable data, we set the target sample to a minimum of 600 participants. A total of 675 participants started the survey, and the final sample included 622 participants, who completed the survey and met eligibility criteria.

### Data collection

Participants were invited via CloudResearch to complete an online survey administered through Qualtrics. Upon accessing the survey, participants first encountered an electronic informed consent form. To proceed, each participant was required to indicate consent by selecting the agree radio button; those who chose disagree were automatically excluded from the survey. Participants were compensated $4.00 for their time.

### Measures

We collected socio-demographic data, including age (years), race, ethnicity, education, income, parenting status, sexual orientation, marital status, health insurance, hometown size, and state of residence.

The Daily Sessions, Frequency, Age of Onset, Quantity of Cannabis Use Inventory (DFAQ-CU) is an assessment tool that is used to measure multiple aspects of cannabis use behavior. It offers a more comprehensive view of cannabis consumption than single-question measures of frequency or quantity. (Cuttler and Spradlin 2017) We adapted items about frequency of use, method of use, and reasons for use to evaluate prenatal cannabis use by referencing pregnancy. Additionally, we modified frequency of use items to assess current and prenatal alcohol and tobacco use.

We used four items from the Marijuana Use Survey (Ng et al. 2022) to assess perceived risks of prenatal cannabis use (i.e., “Cannabis during pregnancy can be harmful to the baby,” “Cannabis during pregnancy can hurt the growth of the baby,” “Using cannabis during pregnancy can cause early birth (preterm),” and “Using cannabis during pregnancy can hurt the child’s ability to learn”). Participants indicated their level of agreement on a five-point Likert scale from *strongly disagree* to *strongly agree*. Higher scores represented greater perceived harm (α = 0.931). We adapted items to assess risk perception of prenatal alcohol (α = 0.916) and tobacco (α = 0.920) use by inserting the respective substance into the statement in place of cannabis.

Participants responded to three items about the safety of cannabis, alcohol, and tobacco use during pregnancy (“How safe do you think it is to use cannabis/alcohol/tobacco when pregnant?”). Participants indicated their perceptions of safety on a five-point Likert scale to determine whether they thought cannabis, alcohol, and tobacco were safe to use during pregnancy, where 1 = *not at all saf*e, and 5 = *completely safe*. There was also an *unsure* response option.

### Analysis

We used descriptive statistics to summarize participants’ demographic characteristics and patterns of cannabis, alcohol, and tobacco use. For participants who had never been pregnant, we conducted pairwise McNemar’s tests to compare 1) willingness to use cannabis products during pregnancy to willingness to use alcohol during pregnancy, 2) willingness to use cannabis products during pregnancy to willingness to use tobacco during pregnancy, and 3) willingness to use alcohol during pregnancy to willingness to use tobacco during pregnancy. We compared proportions of prenatal cannabis, alcohol, and tobacco use among respondents with pregnancy experience. To examine perceptions of safety and risk, we performed repeated-measures Analysis of Variance (ANOVA) using substance type (cannabis, alcohol, tobacco) as the within-subjects factor. We applied Greenhouse–Geisser corrections because the assumption of sphericity was violated for both perceived risk (χ2(2) = 209.369, *p* < 0.001) and perceived safety (χ2(2) = 375.261, *p* < 0.001). To assess effect size, we calculated η^2^ for main effects. We conducted post-hoc pairwise comparisons with adjusted *p* values to evaluate differences between substances and used Cohen’s d to estimate the effect size of post hoc pairwise comparisons.

## Results

### Socio-demographic characteristics

A total of 675 participants started the survey. However, 47 participants did not complete the survey. We excluded from data analysis six participants who reported their age as 16 or 17 years. The final sample comprised 622 participants. The average age was 29.47 years (*SD* = 6.83, range: 18–42). Participants self-identified as White (65.9%, *n* = 410), Black or African American 28.1% (*n* = 175), Asian 5.9% (*n* = 37), American Indian or Alaska Native 3.9% (*n* = 24), or Other 4.7% (*n* = 29); 17.7% (*n* = 110) identified as Hispanic or Latino. Most participants identified as heterosexual (82.0%, *n* = 510). In terms of relationship status, 52.9% (*n* = 329) of participants reported being in a committed relationship or married. The median educational attainment was “Some college,” and the median annual household income as between $40,000–$49,999. Nearly 85% (*n* = 528) reported having health insurance, with the majority (45%, *n* = 237) insured through Medicaid. Many participants, 34.7% (*n* = 216) lived in suburban metropolitan areas, 18.0% (*n* = 112) lived in rural areas, and 15.9% (*n* = 99) lived in large cities with populations over 250,000 people. All regions of the United States were represented; only six states and one territory had no participants (i.e., Alaska, Montana, New Mexico, North Dakota, Puerto Rico, and Vermont). (Table 1).

**Table 1:** Characteristics of participants (*N* = 622)

Characteristic	n	%
**Race**		
American Indian or Alaskan Native	24	3.9
Asian	37	5.9
Black or African American	175	28.1
Native Hawaiian or Other Pacific Islander	2	0.3
More than one race	68	11.0
White	410	65.9
Other (e.g., Brazilian, Multiracial)	29	4.7
**Ethnicity**		
Hispanic or Latino	110	17.7
Not Hispanic or Latino	491	79.2
Other (Asian, mixed, etc.)	8	1.6
**Sexual Orientation**		
Straight/Heterosexual	510	82.0
Lesbian/Homosexual	33	5.3
Bisexual	83	13.3
Other (e.g., pansexual, prefer not to say)	6	0.9
**Health Insurance**		
Not insured	93	15.0
Direct purchase through marketplace (Affordable Care Act)	35	6.6
Employer-sponsored coverage	170	32.3
Medicaid	237	45.0
Medicare	45	8.5
Not sure/other	40	7.6
**Relationship Status**		
Single	261	42.0
In a committed relationship	174	28.0
Married	155	24.9
Separated	8	1.3
Divorced	27	4.3
Widowed	1	0.2
Other/Prefer not to answer	4	0.8
**Parenting Status**		
Have children	337	54.1
No children	285	45.8
**Educational Attainment**		
Never attended school	8	1.3
High school graduate or GED	236	37.9
Some college	212	34.1
College graduate	122	19.6
Graduate/professional degree	31	5.0
Other (e.g., 11th grade, Associate’s degree, certificate)	13	2.1
**Annual Income**		
< $10,000	93	15.0
$10,000–$19,999	47	7.6
$20,000–$29,999	111	17.8
$30,000–$39,999	85	13.7
$40,000–$49,999	47	7.6
$50,000–$59,999	52	8.4
$60,000–$69,999	41	6.6
$70,000–$79,999	42	6.8
$80,000–$89,999	23	3.7
$90,000–$99,999	26	4.2
$100,000–$149,999	41	6.6
> $150,000	14	2.3
**Residential Location**		
Large city (> 250,000)	99	15.9
Medium-sized city (100,000–250,000)	42	6.8
Small city (50,000–99,999)	72	11.6
Suburban (metropolitan area)	216	34.7
Town (< 50,000, not part of city)	81	13.0
Rural (unincorporated area)	112	18.0

### General cannabis, alcohol, and tobacco use

Of the 622 participants, 79.4% (*n* = 494) reported having used cannabis at least once in their lifetime, and 80.2% (*n* = 396) of those had used cannabis in the past year. Of those who reported having used cannabis at least once in their lifetime, 46% resided in states where cannabis was fully legal, 19% of participants resided in states where cannabis was legal for medicinal use only, 19% resided in states where cannabis product use was fully illegal; 16% of participants resided in states where only cannabidiol (CBD) oil was permitted for medical use.

Concerning alcohol, 82.6% (*n* = 514) of the sample had used alcohol at least once in their lifetime, with 82.5% (*n* = 532) of those having consumed alcohol in the past year. For tobacco, 50.5% (*n* = 314) had used tobacco products at least once in their lifetime, with 80.3% (*n* = 499) of those having used tobacco in the past year.

### Cannabis, alcohol, and tobacco use during pregnancy

Of the 352 participants who were currently or had been pregnant, 91 participants (25.9%; 95% CI: 20.81% to 31.74%) reported using cannabis during pregnancy. Of the participants who used cannabis while pregnant, 58.6% resided in states where cannabis was fully legal, 20% of participants resided in states where cannabis was legal for medicinal use only, 16.3% of participants resided in states where only cannabidiol (CBD) oil was permitted for medical use, and 5% participants resided in states where cannabis was fully illegal.

In comparison, 29 (9.5%; 95% CI: 5.51% to 11.83%) reported drinking alcohol while pregnant, and 83 (23.6%; 95% CI: 18.78% to 29.23%) reported using tobacco products while pregnant. The percent of participants who used cannabis products during pregnancy was significantly higher than the percent of participants who used alcohol during pregnancy (χ2 (1, N = 352) = 32.45, *p* < 0.0001), but it did not statistically differ from the percent of participants who used tobacco during pregnancy (χ2 (1, *N* = 352) = 0.50, *p* = 0.544). The percent of participants who used tobacco during pregnancy was also significantly higher than the percent of participants who used alcohol during pregnancy (χ2 (1, *N* = 352) = 25.30, *p* < 0.0001).

Of those who did report cannabis use during pregnancy, the median frequency of use was “Twice a week” (Table 2). Method of cannabis product use varied, joints and blunts were the most common method (Table 3). The most frequent reasons cited for cannabis use during pregnancy included decreasing nausea, reducing anxiety or stress, helping with sleep, relaxing, and reducing pain (Table 4).

**Table 2:** Frequency of cannabis use in pregnancy

Frequency	*n*	%
Once	12	13.19
Once every 3–6 months (2–3 times)	10	10.99
Once every 2 months (4–5 times)	6	6.59
Once a month	3	3.30
2–3 times a month	7	7.69
Once a week	2	2.20
Twice a week	6	6.59
3–4 times a week	11	12.09
5–6 times a week	2	2.20
Once a day	13	14.29
More than once a day	18	19.78

**Table 3:** Method of cannabis use in pregnancy

Method of Cannabis Use	*n*	%
Joint	29	31.87
Blunt (cigar-sized joint)	37	40.66
Hand pipe	4	4.40
Bong (water pipe)	19	20.88
Hookah	4	4.40
Vaporizer (e.g., Volcano, Vape pen)	14	15.38
Edibles	20	21.98

**Table 4:** Reasons for cannabis use in pregnancy

Reason for Prenatal Cannabis Use	*n*	%
Recreation	13	10.99
Decrease nausea	67	73.63
Help with sleep	47	51.65
Reduce anxiety or stress	57	62.64
Reduce pain	43	47.25
To relax	44	48.35
Help with appetite	4	4.40
Recommended by health care provider	9	9.89
Recommended by friend	8	8.79
Did not know they were pregnant	2	2.20

Among participants who had never been pregnant, significantly more participants reported willingness to use cannabis (19.6%, *n* = 53) than alcohol (5.2%, *n* = 14) while pregnant (χ^2^(1, *N* = 269) = 60.36, *p* < 0.001). Additionally, participants were more willing to use cannabis (19.6%, *n* = 53) than tobacco (6.4%, *n* = 17) (χ^2^(1, *N* = 269) = 36.23, *p* < 0.001). All pairwise differences remained significant after we applied a Bonferroni correction (adjusted α = 0.017).

### Perceived risk or safety

We conducted a repeated-measures ANOVA to compare participants’ perceived safety of using cannabis, alcohol, and tobacco during pregnancy (Table 5). The within-subjects effect of substance was significant, indicating that perceived safety ratings varied across substances, with a large effect size (η2 = 0.301). Participants rated cannabis as significantly safer than both tobacco (*p* < 0.001; *d* = 0.670) and alcohol (*p* < 0.001; *d* = 0.701). Tobacco was also perceived as significantly safer than alcohol (*p* = 0.028; *d* = 0.099).

**Table 5:** Perceived safety and risk of using cannabis, alcohol, and tobacco during pregnancy

Variable	Cannabis		Alcohol		Tobacco		*F*	*p*	η2
	*M*	*SD*	*M*	*SD*	*M*	*SD*			
Perceived Safety	3.85	1.46	4.86	0.54	4.79	0.63	212.91	<.001	0.301
Perceived Risk	3.55	1.14	4.48	0.84	4.24	0.96	246.23	<.001	0.285

Note: Higher scores indicate higher perceived harm

For the perceived safety questions, participants could select an “unsure” response option. The percentage of participants unsure about the safety of prenatal cannabis use (16.9%; 95% CI: 13.9% to 20.5%) was significantly different from that of the participants unsure about the safety of prenatal alcohol use (2.3%; 95% CI: 1.2% to 3.8%; χ2 (1, *N* = 622) = 76.33; *p* < 0.001) and prenatal tobacco use (3.5%; 95% CI: 2.2% to 5.4%; χ2 (1, *N* = 622) = 60.12, *p* < 0.001).

All participants reported their perceived risk of adverse effects on fetal development, birth outcomes, and infant development from cannabis, alcohol, and tobacco use during pregnancy. A repeated-measures ANOVA was conducted to compare participants’ perceived risk ratings across substances. The within-subjects effect of substance was significant and a large effect size (η2 = 0.285). Post hoc pairwise comparisons showed that participants rated cannabis as significantly less likely to have adverse effects on fetal development, birth outcomes, and infant development than both tobacco (*p* < 0.001; *d* = 0.596) and alcohol (*p* < 0.001; *d* = 0.734). Tobacco was also perceived as significantly less risky than alcohol (*p* < 0.001; *d* = 0.312) (Table 6).

**Table 6:** Perceived safety of substance use during pregnancy (*N* = 622)

Response Option	Cannabis *n*, %		Alcohol *n*, %		Tobacco *n*, %	
Completely safe	61	9.8%	2	0.3%	5	0.8%
Mostly safe	55	8.8%	9	1.4%	6	1.0%
Somewhat safe	68	10.9%	16	2.6%	22	3.5%
A little safe	62	10.0%	13	2.1%	38	6.1%
Not at all safe	269	43.2%	568	91.3%	528	84.9%
Unsure	105	16.9%	14	2.3%	22	3.5%

## Discussion

Our study offers valuable insights into cannabis use and perceptions of the risks linked to cannabis, alcohol, and tobacco during pregnancy among a reasonably sized sample of U.S. women of childbearing age. Although our sample differed from the broader U.S. population in terms of educational attainment and socioeconomic status, several notable patterns emerged. Participants consistently viewed cannabis as safer, more acceptable, and more commonly used during pregnancy than either alcohol or tobacco. We also observed that cannabis use varied by state legal status, with the highest prevalence reported among participants living in states where recreational cannabis was fully legal. However, cannabis use was not confined to these states, as 16–19% of participants who reported use resided in states where cannabis was legal only for medical use, where only cannabidiol (CBD) was permitted for medical purposes, or where cannabis remained fully illegal.

Participants who had not been pregnant perceived cannabis to be less harmful or more acceptable than alcohol or tobacco. Among participants with histories of pregnancy, more than one-fourth reported prenatal cannabis use, substantially higher than national surveillance estimates, which generally suggest cannabis use prevalence between approximately 4–10%. (Ko et al. 2015) Use was frequent, and approximately half of the participants reported consumption several times per week, and inhalation via joints or blunts emerged as the predominant mode of administration.

Safety and risk perceptions highlight cultural shifts in attitudes and beliefs about cannabis. Participants rated cannabis as safer than tobacco and alcohol, and they reported higher uncertainty about the risks of cannabis than for the other substances. This pattern has been observed in earlier studies, where pregnant individuals described cannabis as a more natural and potentially safer option than alcohol, tobacco, or certain medications during pregnancy. (Jarlenski et al. 2016) Participants’ reasons for use, including nausea relief, stress reduction, and sleep improvement, align with other researchers’ findings that cannabis is used for therapeutic reasons and not only for recreation. (Lu and Mackie 2021) Further evidence from a recent statewide study in Arizona is consistent with the idea that many budtenders recommend cannabis products to pregnant women looking for relief from morning sickness. (Madson et al. 2025).

These attitudes and behaviors are concerning, given emerging evidence of potential harms. Prenatal cannabis exposure has been linked to impaired fetal growth and altered brain development. (Etemadi-Aleagha and Akhgari 2022) The frequency of use reported in our study among pregnant participants, typically twice per week and, in some cases, daily, may heighten risks during sensitive periods of neurodevelopment. DeGenna et al. found that PCE produced nuanced, long-lasting effects on memory and achievement in children and adults. Further, they found PCE to be consistently linked to externalizing outcomes, including delinquent behavior and substance use, that extend into adult years. (Genna et al. 2022) In contrast, alcohol and tobacco were used more sporadically during pregnancy, suggesting that cannabis may represent a qualitatively different exposure profile.

The broader social context may influence these patterns. While alcohol and tobacco have been the focus of decades of public health efforts, communication about cannabis remains inconsistent and is often framed through harm-reduction or pro-cannabis narratives, especially on social media platforms. (Vogel et al. 2025) Along with increasing legalization and normalization, such messaging is likely to shape perceptions of safety. Our study findings highlight how women assess the relative risks of different substances and often choose cannabis over alcohol or tobacco because they view it as less harmful.

### Limitations

While the detailed survey is a strength, certain limitations are acknowledged. The sample was moderate in size, not representative of the entire population, and online data collection restricted participation. The data were self-reported and retrospective, making them vulnerable to recall bias and social desirability bias, especially concerning sensitive topics like substance use during pregnancy. The cross-sectional design also restricts causal inferences. Additionally, factors such as medical versus recreational use, the source of cannabis products, and education on managing symptoms during pregnancy were not thoroughly explored but could substantially influence risks and decision-making. We also did not track the number of pregnancies or specify which pregnancies participants used cannabis, alcohol, or tobacco in. Perceived risks and safety perceptions of substance use may vary across different pregnancies based on prior experiences. Furthermore, our sample size was insufficient to collect data on polysubstance use during pregnancy or to analyze whether attitudes differ by demographic factors or specific substance use during pregnancy. Our findings are also limited to the United States, and perceptions may vary in different cultures and countries. These areas are important for future research.

### Implications

Our study findings underscore the urgent need for evidence-based public health communication about cannabis use during pregnancy. At the policy level, expanding legalization and commercialization of cannabis should be accompanied by clear, evidence-informed maternal health warnings that parallel those used for alcohol and tobacco. Such warnings should be standardized, prominently displayed on product packaging and at points of sale, and explicitly address pregnancy-related risks, uncertainty, and the lack of established safety thresholds. Policies could also require cannabis retailers to provide consistent, non-misleading information and prohibit marketing claims that frame cannabis as a safe or therapeutic option during pregnancy. Integrating these warnings into public health campaigns, prenatal care settings, and digital platforms would help counteract normalization narratives and ensure that pregnant individuals receive accurate, accessible information to support informed decision-making.

## Conclusion

As social acceptance grows, especially in states where recreational or medical cannabis is legal, misconceptions about its safety could increase prenatal exposure. The common perception of cannabis as therapeutic, harmless, or natural calls for a tailored public health approach that respects individual choices while providing clear, evidence-based information. Moving forward, effective strategies must include accurate messaging and account for the social, cultural, and emotional factors that influence substance use during pregnancy. Addressing these issues is essential to protect maternal and fetal health and ensure informed, fair care.

## Data Availability

The dataset collected and analyzed in the current study is available from the corresponding author upon reasonable request.
